# Food safety knowledge and practices among raw meat handlers and the microbial content of raw meat sold at Kumasi Abattoir Butchery Shops in Kumasi, Ghana

**DOI:** 10.1186/s12889-024-18514-w

**Published:** 2024-04-08

**Authors:** Desmond Azeko Asati, Prosper Manu Abdulai, Kofi Sekyere Boateng, Abigail Abena Anokyewaa Appau, Linda Aurelia Ofori, Thomas Peprah Agyekum

**Affiliations:** 1 Department of Occupational and Environmental Health and Safety, School of Public Health, College of Health Sciences, Kwame Nkrumah University of Science and Technology, Kumasi, Ghana; 2 Department of Public Health Education, Akenten Appiah-Menka University of Skills Training and Entrepreneurship, Asante Mampong, Ghana; 3 Department of Theoretical and Applied Biology, Faculty of Biosciences, College of Science, Kwame Nkrumah University of Science and Technology, Kumasi, Ghana

**Keywords:** Food safety, Foodborne illness, Abattoir, Meat safety, Meat handler, Slaughter stock

## Abstract

**Background:**

Foodborne diseases affect nearly 600 million people each year, that is, one in every ten people, and their outbreaks are most common in low- and middle-income countries, particularly in Africa. This study investigated the food safety practices among raw meat handlers and the microbial quality of the meat from the butchery shops in Kumasi Abattoir, Ghana.

**Methods:**

This study employed a descriptive cross-sectional study and collected quantitative data on factors associated with food safety and hygienic practices among raw meat handlers and the microbial quality of the raw meat using a structured questionnaire and standard laboratory methods, respectively. The study used all 50 beef vending shops in the butchery for questionnaire aspect and fresh beef samples were obtained from 10 vendors in the butchery shop. Appropriate methods were followed to analyse questionnaire data and meat samples.

**Results:**

Most of the butchers (72%) were between the ages of 31 and 45, and they were predominantly Muslims (68%). Most of the respondents (48%) had basic education. All the respondents had food safety certificates from the local authority but needed adequate knowledge of meat safety. Most respondents (90%) handled meat and money with the same bare hands, thus contaminating the meat. The study showed that the maximum Total Viable Count (TVC), Total *Staphylococcus* Count (TSC), and Total *Escherichia coli* Count (TEC) were 5.60, 4.39 and 5.13 cfu/g, respectively. The study also revealed that all the meat samples were *Salmonella* species-free.

**Conclusions:**

Microorganisms in raw beef indicate a public health hazard. It gives a signal of a possible occurrence of food-borne intoxication and infection if not controlled. Environmental health officers in the Greater Kumasi area should organize food safety training and educate raw meat handlers on the importance of food safety and its consequences.

## Introduction

Food safety has been increasingly important to consumers as civilization has progressed. Many countries are increasingly interdependent on the availability of their food supply and on its safety [1]. Thus, food safety is of key importance to the survival of humans and the development of countries [2]. Food safety is directly affected by highly poisonous pesticides, hazardous chemical substances, microbiological contamination, and food producers’ unhygienic practices [3]. Foodborne diseases harm nearly 600 million people each year, that is, one in every ten people. Children under the age of five are especially vulnerable, with 125,000 of them dying each year due to eating contaminated food, accounting for 40% of all foodborne mortality [4]. According to the World Health Organization (WHO), the African Region bears the most significant load of per capita foodborne infections. It is projected that over 91 million people are anticipated to fall ill each year, resulting in around 137,000 fatalities [5].

Foodborne outbreaks are most prevalent in low- and middle-income nations and are closely associated with inadequate food handling and sanitation practices, insufficient food safety regulations, inefficient regulatory systems, limited financial resources for implementing safer equipment, and a lack of education among food handlers [6]. These diseases of food origin can cause short-term symptoms, such as nausea, vomiting, and diarrhea, termed food poisoning, and chronic conditions like kidney failure, cancer, liver failure, and brain and neurological disorders [4]. Children, pregnant women, and individuals who are elderly or have a compromised immune system may be especially vulnerable to these infections [4]. Children who survive some of the most serious foodborne illnesses may experience delays in physical and mental development, which can have a long-term impact on their quality of life [4]. *Salmonella*, *Campylobacter*, and *Escherichia coli* (*E. coli* O157:H7) are the most prevalent bacterial agents causing foodborne illnesses. *Salmonella* and *E. coli* O157:H7 are reported to cause approximately 1.3 million and 62,000 cases of gastroenteritis, respectively, in the United States of America each year [7].

In Ghana, several studies have investigated food safety practices among street vendors and other institutional centres [8–13]. Despite the significant contribution of raw meat handlers to increasing foodborne diseases across the globe [14, 15], much research attention has not been given to this area. Health authorities and the healthcare system in Ghana face a notable health challenge due to foodborne illnesses like Cholera, Typhoid fever, Dysentery, and Viral Hepatitis [16]. These ailments can result from contact with tainted food and unclean water, exacerbated by inadequate hygiene, limited literacy, and educational levels [17].

Meat is one of the rich protein sources for man, being the source of several vitamins, especially vitamin A and B12, which cannot be obtained from plant sources [18]. Human foods from animal sources are the most exposed to hazards and are often regarded as high-risk commodities for pathogen content, natural toxins and many other contaminants. This contamination is partly due to the processing and handling of meat and its products [4, 18].

The demand for meat is rising due to rising populations, urbanization, high disposable incomes and changing eating habits [19]. This increasing demand must be met with increased supply from the meat markets – abattoirs and butchery shops. This also comes with health risks and hazards if meat preparation is not done under hygienic conditions. Therefore, there is a need to adopt and practice food safety practices to eliminate or minimise contamination of meat and meat products. The most effective way to reduce disease transmission from food handlers to consumers is to practice excellent meat hygiene in private and professional settings.

Although consumer awareness and demand for food safety standards, including meat, have recently been on the rise, there are still serious concerns about personal hygiene and sanitary practices, leading to potentially unacceptable levels of microbes in meat [20]. Meat products quickly cause foodborne illnesses because of the susceptibility of meat to contamination, mainly through the processing and handling stages. Despite this high risk, meat is a delicacy and an excellent source of protein for humans. Meat consumption has risen in recent years [21], and its safety has become a serious public health concern. In Ghana and most developing countries, the systems for ensuring food safety still need to be fully implemented and practised. A system such as the Hazard Analysis Critical Control Points (HACCP) must be better implemented in Ghana [22]. Thus, this study evaluated the food safety practices employed by individuals handling raw meat and examined the microbial quality of the meat sold in the butchery shops in Kumasi Abattoir, Ghana.

## Methods

### Study area

**Fig. 1:**
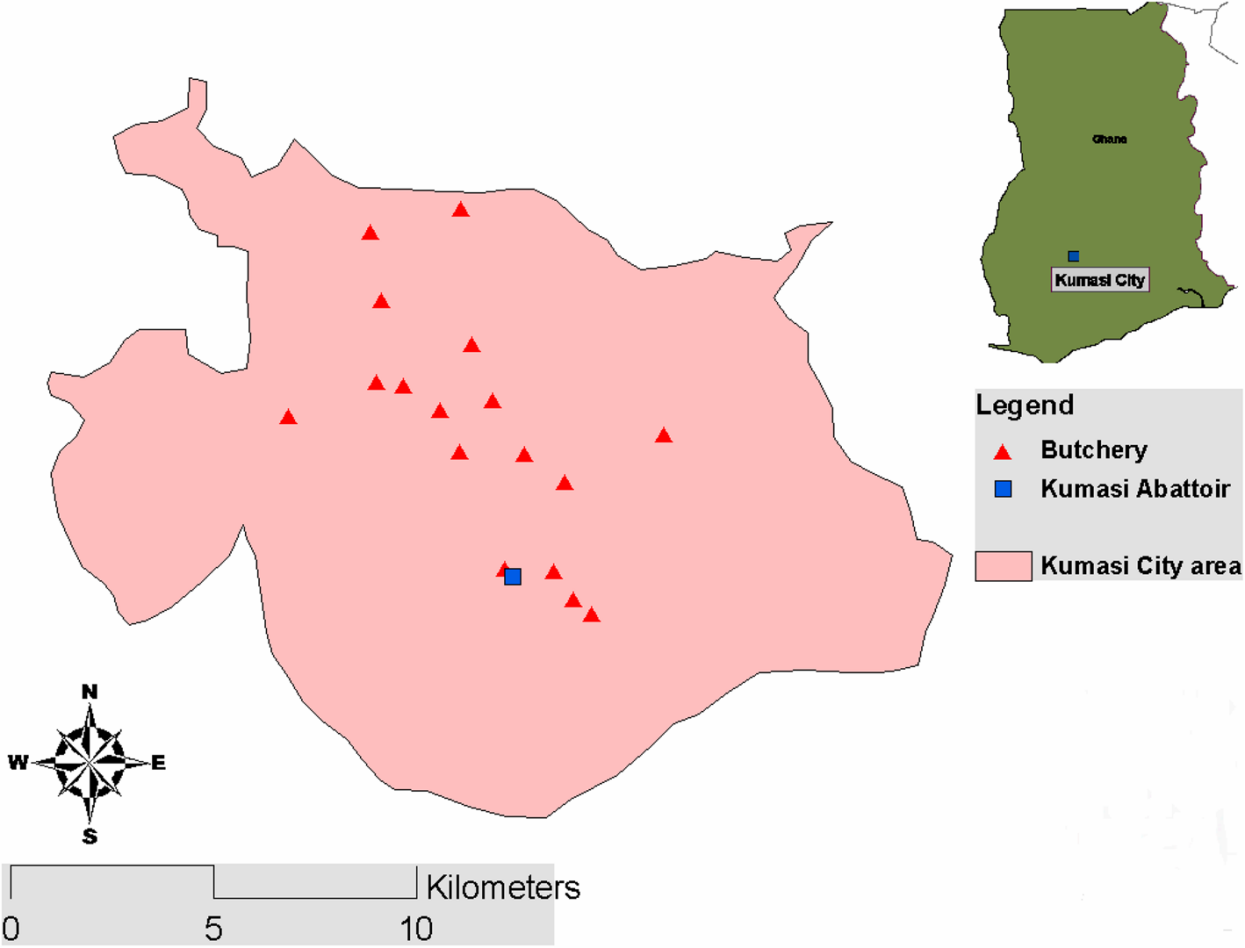
Map of Kumasi showing the Kumasi Abattoir and other butcheries within the city

The study was conducted in the Kumasi Abattoir (coordinates 6˚39’36.6"N Latitude and 1˚36’15.4"W Longitude) in Kumasi Metropolis, Ashanti Region of Ghana. The Abattoir is operated by the Kumasi Abattoir Company Limited (KACL) and comprises a slaughterhouse and a butchery that houses about 100 raw meat sellers. It also supplies meat to other private butcher shop owners in the city, including the Kejetia meat shop. The Kumasi Abattoir Company Limited (KACL) has been a central facility for meat production and processing since 1999, adhering to high standards of quality, safety, and health recognized globally. Many local butchers receive meat from the Kumasi abattoir (Fig. 1) [23]. The study was conducted in two sections: interviewing meat dealers and taking meat samples for microbiological assessment.

### Questionnaire section

#### Study population

The study population consisted of raw meat handlers working at the Kumasi Abattoir butchery. The beef vending shops in the butchery were about 50 with the conditions under which each operates being significantly different, however, vendors in a particular shop work under the same conditions. We therefore selected one meat handler from all 50 shops.

#### Eligibility criteria

The study included meat handlers who have worked with fresh meat for at least one year, meat vendors affiliated with a beef vending shop in the butchery, and respondents in these two categories who provided verbal/written consent to participate. However, we excluded those who did not meet these requirements, meat handlers, and vendors who were not available at the time of the study. Illiterate participants were not involved in this study.

#### Sampling methods/techniques and sample size

A purposive sampling method was used to select meat handlers and vendors at the Kumasi Abattoir. The abattoir has about 50 butchery shops dealing in beef. Thus, we selected a representative from the 50 shops for questionnaire administration. When more than one meat handler or vendor from a shop was willing to participate in the study, the person who spent more time handling meat was selected and interviewed. One questionnaire was administered to one worker at a shop.

#### Data collection tools and techniques

The study employed a structured closed-ended questionnaire adapted from Gomes-Neves [24] with some modifications. The questionnaire was structured into four sections: The first section assessed the socio-demographic information of the meat handlers. Data such as age, gender, education level, job position, and years of service were obtained. Section B evaluated meat handlers’ food safety knowledge, Section C examined personal hygiene practices, and Section D focused on food safety practices.

#### Pretesting of questionnaire

Pretesting of the questionnaire was done among raw meat sellers at Kejetia market in Kumasi, Ashanti Region. Ten meat vendors were selected for the pretesting. The setting of the Kejetia market butchery shops has similar characteristics to that of the Kumasi abattoir butchery. These include the dynamic nature of the area, the population of people patronizing the site, the design and arrangement of the shops, the source of meat and so on. The necessary modifications were made to the questionnaire after pretesting to ensure they addressed the study objectives.

#### Ethical approval

The study received ethical approval from the Kwame Nkrumah University of Science and Technology (KNUST) Ethical Review Committee (CHRPE/AP/372/23), and permission to conduct the study was obtained from the Kumasi Abattoir Company Limited. The individual meat handlers who participated in the study provided written consent. The raw meat handlers were not identified by name but were assigned codes.

### Microbiological assessment of meat samples

#### Meat samples collection

Fresh beef samples were obtained from ten different vendors in the butchery shop of the Kumasi Abattoir. Specifically, the study focused on freshly cut beefsteaks obtained from the fore or hind limb regions for sampling purposes. Each sample weighed 100 g and was collected with strict adherence to hygienic practices using sterile polythene pouches [15]. The pouches were then sealed and immediately placed on ice for transportation to the KNUST Microbiological Laboratory. This process was conducted within a few hours of collection to ensure the freshness of the samples. The procedure was repeated weekly for three weeks in January 2022, collecting thirty fresh samples for further microbiological analysis.

#### Chemical reagents

The agars utilised came from OXOID Laboratories, England, UK. These agars included Plate Count Agar (PCA) for determining the total viable count, Brilliance *E. coli* Agar (BEA) for isolating *Escherichia coli*, Brilliant Green Agar (BGA) and Rappaport Vassiliadis Broth (RVB) for isolating *Salmonella*, and Mannitol Salt Agar (MSA) for isolating *Staphylococcus*.

#### Media preparation

All media; Plate Count Agar, Mannitol Salt Agar, Rappaport Vassiliadis Broth, Xylose Lysine Deoxycholate, Brilliant Green Agar and Brilliance *E. coli* Agar were prepared according to manufacturer’s instructions. The right amount as prescribed was weighed into its respective volume of water and autoclaved at 121 °C for 15 min before use.

#### Meat sample preparation

Ten grams of shredded beef samples were carefully placed in a clean, sterilised stomacher bag with 90 ml of peptone water was added and pulsified for 15 s using a pulsifier. One millilitre of the homogenous mixture was poured into a test tube containing 9 ml of distilled water to achieve a 10^−1^ dilution. The mixture was then shaken well using a vortex mixer, and a series of dilutions up to 10^−4^ were created for microbiological analysis.

#### Microbiological analysis

The methods outlined subsequently were employed to detect the existence of microorganisms in beef. The colonies on specific plates were quantified using a colony counter, and the physical characteristics of the colony, such as colour, shape, and size, were analysed to aid in grouping and identification.

##### Diluent preparation and serial dilution

This study utilized buffered peptone water from Biolab Scientific Limited, Canada as the diluent. It was prepared per the manufacturer’s guidelines on the label. The initial dilution was made by mixing 10 g of the sample with 90 ml of sterile diluent and shaking it for 30 s. Further dilutions were then created by adding 1 ml of the stock solution to 9 ml of sterile diluent step-by-step.

##### Total Viable Count (TVC)

The Total Viable Counts were determined by spreading a diluted sample on Plate Count Agar, incubating it at 37 °C for 24 h, and then counting the number of colonies that formed [25]. The sample was first diluted by mixing 10 g with 90 ml of sterilized peptone water and shaking for 15 s. Then, 1 ml aliquots from each dilution were placed on pre-prepared Petri dishes with Plate Count Agar (PCA). The plates were then inverted and incubated for 24 h at 37 °C. The colonies that formed were enumerated and recorded as the Total Viable Count.

##### Enumeration of *Staphylococcus* species

The spread plate method was used to isolate and count *Staphylococcus* species grown on Salt Mannitol Agar [26]. Dilutions of the sample, ranging from 10^−1^ to 10^−4^, by mixing 10 g of the sample with 90 ml of sterilized peptone water and pulsifying for 15 s. 1 ml portion from each dilution was dispensed onto Petri dishes filled with prepared Salt Mannitol Agar (SMA). A sterile bent rod was used to evenly spread the samples, leaving them to air-dry for 15 min at room temperature. Subsequently, the plates were incubated at 35 ºC for 24 h. Following the incubation, the number of yellow colonies was tallied and documented as the *Staphylococcus* count, utilizing a colony counter.

##### Enumeration of *Escherichia coli*

The pour plate method detected and enumerated *E. coli* in the samples [26]. The samples were cultured on MacConkey Agar (MA). To create dilutions of the sample, ranging from 10^−1^ to 10^−4^, 10 g were mixed with 90 ml of sterilized peptone water and pulsified for 15 s. Subsequently, 1 ml from each dilution was plated onto Petri dishes containing pre-made MacConkey agar. The plates were then incubated at 37 ºC for 24 h. Following the incubation period, the number of pink colonies was enumerated and documented as the *E. coli* count with the help of a colony counter. To finally confirm *E. coli*, the pink colonies were cultured on a highly selective media, Brilliance *E. coli* Agar, to observe distinct purple colonies.

##### Enumeration of *Salmonella*

The Brilliant Green agar (BGA) and Xylose Lysine Deoxycholate agar (XLD) were the media used, and the preparation was done according to the manufacturer’s instructions. The agar plates were prepared and sterilized overnight before the test.

In the pre-enrichment phase, 1 ml of the stock dilution is mixed with 9 ml of 10% bacteriological peptone and incubated for 24 h at 37^o^C. In the next stage, 100 µL of the peptone-sample mixture is added to 9 ml of Rappaport Vassiliadis Broth (RVB) and incubated for another 24 h. The final phase is where 10 µL of the RVB is added to BGA and XLD agar plates and incubated for 24 h. The presence of red colonies with black canters was observed afterwards.

#### Quality control and assurance

The microbial analyses were also done using standard laboratory methods. A qualified laboratory technician helped with the analysis at the microbiology laboratory to ensure the accuracy of findings.

### Statistical analysis

The data processing and analysis for the study were performed using Statistical Package for the Social Sciences (SPSS) software (version 23.0). The SPSS was utilized to analyze the data collected through the questionnaire. Before entry, the responses to the questions were coded. Descriptive statistics, including tables and percentages, were employed to analyze most of the variables in the study. The data obtained from the microbiological analysis of the carcasses was initially transferred to Microsoft Excel 2016 in its raw format and exported into SPSS for statistical analysis. To achieve a normal distribution, the data were transformed into log_10_ values. A one-way analysis of variance (ANOVA) with the Tukey posthoc test was conducted to assess the differences in the microbial species’ results among the shops. A significance level of *P <* 0.05 was considered significant.

## Results

### Demographic information

All the respondents were males, and most (72%) were between 31 and 45 years old. Regarding religion, 32% of the respondents were Christians, while 68% were Muslims. Most respondents had basic (48%) and secondary (38%) education, with only 4% having attained tertiary education. All of the respondents had a food safety certificate. Five respondents (representing 10%) were workers, while 45(90%) of them were owners of the shops/businesses (Table 1). Concerning work experience, most of the respondents (80%) had worked for more than ten years. The majority of the respondents are from Frafra (56%), followed by Dagomba respondents (36%), with only 8% being Ewes (Table 1).

**Table 1:** Demographic characteristics of the respondents

Variable	Frequency (N)	Percentage (%)
**Gender**		
Male	50	100
Female	0	0
**Age (in years)**		
18–30	6	
31–45	36	72
46–60	9	18
Above 60	4	
**Religion**		
Christianity	16	32
Islam	34	68
African Traditional	0	0
**Level of Education**		
No formal education	5	10
Basic	24	48
SHS/Vocational	19	38
Tertiary	2	4
**Work Experience (in years)**		
Below 1	0	0
1–5	3	6
6–10	7	14
Above 10	40	80
**Position at work**		
Owner	45	90
Employee	5	10
**Food Safety certificate**		
Yes	50	100
No	0	0
**Ethnicity**		
Dagomba	18	36
Frafra	28	56
Ewe	4	8

### Respondents’ knowledge of food safety

We assessed respondents’ knowledge of food safety by asking nine questions about symptoms of foodborne illnesses, high-risk groups, effects of foodborne illnesses, etc. The results showed that 90% of the respondents said old people, children, and patients with immunodeficiency were most at risk. Six per cent of the respondents selected body pains as a common symptom of food-borne diseases caused by contaminated meat, while 80% of the respondents selected abdominal pains, fever, diarrhoea and vomiting. Respondents were further asked what happens to food contamination when hands are washed before work begins. Most respondents (94%) said food safety issues will be minimised, while 6% said washing hands will not affect food safety. Similarly, 44% of the respondents said food contamination is reduced when handlers use gloves during meat handling, while 56% said it will have no effect. Only 4% of the respondents said food contamination is minimized when no one eats or drinks in the workplace. Two respondents (representing 4%) said it is maximized, while 46 (92%) said it has no effect (Table 2).

**Table 2:** Food safety knowledge of respondents

Variable	Frequency (N)	Percentage (%)
**Groups at high risk of food-borne diseases**		
No one	0	0
Old people, children and patients with immunodeficiency	45	90.0
General public	5	10.0
**Common symptom(s) of food-borne diseases caused by contaminated meat**		
Bleeding	0	0
Body pains	3	6.0
Abdominal pains, fever, diarrhea and vomiting	40	80.0
I do not know	7	14.0
**What happens to food contamination when hands are washed before work begins?**		
Minimized	47	94.0
Maximized	0	0
No effect	3	6.0
**What happens to food contamination when gloves are used during meat handling?**		
Minimized	22	44.0
Maximized	0	0
No effect	28	56.0
**What happens to food contamination when one eats and drinks in the workplace?**		
Minimized	2	4.0
Maximized	2	4.0
No effect	46	92.0
**Can one contract typhoid by eating contaminated meat?**		
Yes	13	26.0
No	7	14.0
Uncertain	30	60.0
**Food-borne diseases can induce abortion in pregnant women**		
True	12	24.0
False	7	14.0
Uncertain	31	62.0
**One needs to take sick leave when he/ she has a skin disease**		
True	50	100.0
False	0	
Uncertain	0	
**Other food items like vegetables can be stored together with the meat in the freezer**		
True	0	0
False	50	100.0
Uncertain	0	0
**Eating meat can cause HIV/AIDS**		
True	0	0
False	47	94.0
Uncertain	3	6.0

Regarding whether one can contract typhoid by eating contaminated meat, most respondents (60%) said they were uncertain, 26% retorted in the affirmative, and 12% said no. When asked if food-borne diseases can induce abortion in pregnant women, 31 respondents (representing 62%) answered that they were uncertain, 24% indicated food-borne diseases can induce abortion in pregnant women, while 14% said it was false. All the respondents agreed that one needs to take sick leave when he/she has a skin disease.

### Food safety practices among raw meat handlers

#### Personal hygiene

**Fig. 2:**
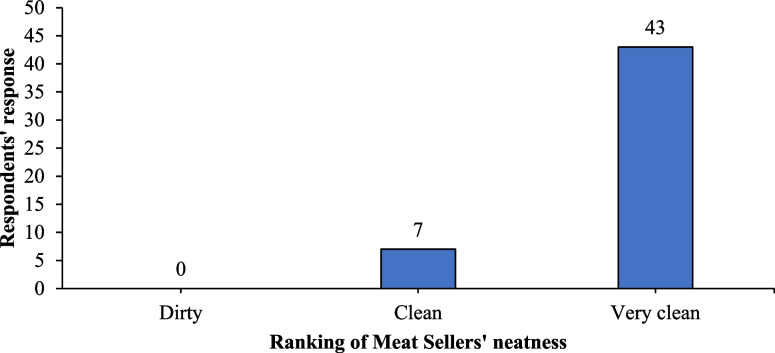
Ranking meat sellers on neatness

Most respondents (42%) did not wear aprons while working. None of them used gloves as they worked. All the respondents affirmed the washing of hands before touching raw meat. Thirty-one respondents, representing 62%, worked without a nose mask (Table 3). Ninety per cent (90%) of the respondents handled meat and money with the same bare hands. All of the respondents had short and clean nails. On ranking meat sellers on their neatness, we observed respondents’ work attire, nails, and their general appearance. Any respondent with clean work attire (without ingrained dirt), short and lean nails and generally appearing clean was ranked as very clean. Any respondent scoring one or two of these was ranked clean while any respondent observed to be lacking in all three was ranked dirty. The study revealed that 43(86%) were ranked as very clean, while 7(14%) were categorised as clean (Fig. 2).

**Table 3:** Observations on personal hygiene of respondents

Statements	Responses	
	Yes	No
Wearing an apron during work	29 (58)	21 (42)
Use of gloves while working	0 (0)	50 (100)
Washing of hands before touching raw meat	50 (100)	0 (0)
Use of a nose mask during work	19 (38)	31 (62)
Wearing a cap during work	21 (42)	29 (58)
Wearing waterproof footwear during	9 (18)	41 (82)
Meat and money are handled by the same person	45 (90)	5 (10)
Nails short and clean	50 (100)	0 (0)
Cleanliness of working clothes	50 (100)	0 (0)

#### Meat safety practices

All respondents said their knives, chopping and cutting boards/tables are washed twice daily (at the beginning and end of work). All respondents affirmed that their hands are always washed before handling meat. Fourteen per cent of the respondents said they sometimes use gloves during work, while 86% said they never use gloves (Table 4). Forty-eight handlers (96%) responded that smoking at the workplace is not a good practice. Less than half (36%) of the respondents stated they sterilize the knives and other equipment. All agreed that the mouth and nose should be covered when sneezing or coughing. Fouty-one (82%) of the respondents were certain that it is not appropriate to rub their hands on their hair or face while working, 14% of respondents said they were uncertain, and 4% said it was appropriate (Table 4).

**Table 4:** Meat safety practices of respondents

Variable	Frequency (N)	Percentage (%)
**Frequency of washing the chopping/cutting tables**		
Several times a day	0	0
At the beginning and end of work	50	100
Once in a week	0	0
**Frequency of washing the knives**		
Several times a day	0	0
At the beginning and end of work	50	100
Once in a week	0	0
**Frequency of hand washing before handling meat**		
Minimized	0	0
Maximized	7	14
No effect	43	86
**Smoking in the workplace is not a good practice**		
Yes	48	96
No	2	4
Uncertain	0	0
**Sterilization of knives**		
Yes	18	36
No	32	64
Uncertain	0	0
**Should the mouth and nose be covered when sneezing or coughing?**		
Yes	50	100
No	0	0
Uncertain	0	0
**Rubbing the hands on the face and hair while working is not appropriate**		
Yes	41	82
No	2	4
Uncertain	7	14
**Wedding rings and watches can be worn while handling meat**		
Yes	2	4
No	10	20
Uncertain	38	76
**The apron can be used to clean dirty hands during meat handling**		
Yes	0	0
No	45	90
Uncertain	5	10
**Meat should not come into contact with a wounded hand/sore**		
Yes	50	100
No	0	0
Uncertain	0	0

### Microbial loads on beef carcasses

The highest total viable count (TVC) (expressed as Log_10_ cfu/g) was recorded at shop number 11 (5.60) and the minimum at shop number 5 (2.85). The results also showed that the highest *Staphylococcus aureus* count (TSC) (expressed as Log_10_ cfu/g) was 4.39, recorded at shop 13 and the lowest at shop 10 (1.74). The maximum *Escherichia coli* count (TEC) was recorded at shop 17 (5.13) and the least at shop 12 (2.31). The study has also revealed that meat samples from all 20 shops were free of the *Salmonella* species. The means TVC, TSC and TEC values were 4.85, 3.13 and 2.96 Log_10_ cfu/g, respectively. The mean counts for TVC, TSC, and TEC in all the meat shops did not exhibit a significant difference (*P <* 0.05). Among the isolated bacterial genera, *Staphylococcus aureus* and *Escherichia coli* had the highest occurrence, accounting for 53% and 47%, respectively, while *Salmonella* species were not found. *Staphylococcus* species were identified in all the meat samples from the various shops, and *Escherichia coli* was present in all the shops except for shops 9 and 10 (Table 5). There was no *Salmonella* count recorded in the meat samples.

**Table 5:** Microbial load of raw beef sold in the Kumasi Meat Abattoir Butchery shops

Shop ID	Average TVC/cfu	Average log TVC	Average TSC/cfu	Average log TSC	Average TEC/cfu	Average log E. coli count
1	4.24$\times$10^4^	4.63	5.75$\times$10^2^	2.76	6.50$\times$10^2^	**2.81**
2	5.95$\times$10^4^	4.77	1.87$\times$10^2^	2.27	4.55$\times$10^2^	**2.66**
3	4.22$\times$10^4^	4.63	6.00$\times$10^2^	2.78	4.05$\times$10^2^	**2.61**
4	8.98$\times$10^3^	3.95	4.05$\times$10^2^	2.61	4.35$\times$10^2^	**2.64**
5	7.05$\times$10^2^	2.85^b^	3.05$\times$10^2^	2.48	2.20$\times$10^2^	**2.34**
6	9.32$\times$10^2^	2.97	1.65$\times$10^2^	2.22	1,55$\times$10^2^	**2.19**
7	5.63$\times$10^4^	4.75	1.04$\times$10^4^	4.02	6.78$\times$10^3^	**3.83**
8	3.13$\times$10^4^	4.50	6.66$\times$10^3^	3.82	5.29$\times$10^3^	**3.72**
9	2.75$\times$10^5^	5.44	9.5$\times$10^1^	1.98	NIL	**NIL**
10	2.01$\times$10^5^	5.30	5.5$\times$10^1^	1.74^b^	NIL	**NIL**
11	3.95$\times$10^5^	5.60^a^	2.55$\times$10^2^	2.41	3.90$\times$10^2^	**2.59**
12	3.47$\times$10^2^	5.54	1.60$\times$10^2^	2.20	2.05$\times$10^2^	**2.31**^**b**^
13	1.93$\times$10^5^	5.29	2.48$\times$10^4^	4.39^a^	9.64$\times$10^3^	**3.98**
14	2.24$\times$10^5^	5.35	1.92$\times$10^4^	4.28	6.93$\times$10^3^	**3.84**
15	2.93$\times$10^4^	4.47	3.34$\times$10^3^	3.52	1.05$\times$10^3^	**3.02**
16	2.30$\times$10^4^	4.36	1.93$\times$10^3^	3.29	8.07$\times$10^2^	**2.91**
17	3.90$\times$10^5^	5.59	2.00$\times$10^4^	4.30	1.36$\times$10^5^	**5.13**^**a**^
18	3.67$\times$10^5^	5.56	2.35$\times$10^4^	4.37	5.91$\times$10^4^	**4.77**
19	5.58$\times$10^5^	5.75	5.44$\times$10^3^	3.74	9.69$\times$10^3^	**3.99**
20	5.05$\times$10^5^	5.70	2.93$\times$10^3^	3.47	6.94$\times$10^3^	**3.84**

Total *Salmonella* counts were all zero so there was no transformation into log

*TVC *Total Viable Count, *TSC *Total Staphylococcus Count, *TEC *Total *Escherichia coli* Count, *TSSC *Total *Salmonella* Count, *a* Maximum, *b *Minimum

### Microorganisms identified in the raw beef samples from the Kumasi Meat Abattoir Butchery shops

*Staphylococcus* species and *Escherichia coli* were the most commonly found bacteria species. No *Salmonella* species were identified in any analysed samples (Table 6).

**Table 6:** Bacteria identified in raw beef in the Kumasi Meat Abattoir Butchery shops

	Type of Bacteria Identified		
Shop ID	*Staphylococcus spp*	*Escherichia coli*	*Salmonella spp*
1	+	+	-
2	+	+	-
3	+	+	-
4	+	+	-
5	+	+	-
6	+	+	-
7	+	+	-
8	+	+	-
9	+	-	-
10	+	-	-
11	+	+	-
12	+	+	-
13	+	+	-
14	+	+	-
15	+	+	-
16	+	+	-
17	+	+	-
18	+	+	-
19	+	+	-
20	+	+	-

NB: **+** Means bacteria is present; - means bacteria is absent

## Discussion

This study investigated food safety knowledge and practices among raw meat handlers and the microbial content of raw meat sold at Kumasi Abattoir Butchery Shops in Kumasi, Ghana. An observational study by Ansari-Lari [27] found that while food handlers with inadequate understanding did not demonstrate food safety in their activities, those with strong knowledge did. The current study’s findings indicated that respondents had sufficient awareness of food safety. The high awareness of food safety could be attributed to the training of respondents on food safety as all of them had certificates in food safety. Food handler training is perceived to be one strategy to create awareness of food safety practice, thus providing long-term benefits to the food handlers and establishments [28]. These findings are similar to those reported by Akabanda [8] among institutional food handlers in Ghana and Makhunga [29] in charitable food assistance programmes in the eThekwini District, South Africa. The effects of eating contaminated food on individuals and the general population are known to all respondents. Only a few respondents claimed to be unaware of the symptoms of consuming contaminated food. The findings of this study agree with research conducted by Tokuç [30] and Yenealem [31] from Ethiopia, who reported that individuals responsible for handling meat understood the significance of personal hygiene practices; however, they failed to implement them. Yenealem [31] however explained that this is the reality of most developing countries, like Ghana, where adequate knowledge does not correlate with practice sometimes because of lack of essential facilities. The results of the current study, however, contradict other studies reported in Africa where food safety knowledge among food handlers was poor [32]. A scoping review [33] conducted in all LMICs (except Nigeria) as per the World Bank Global Index LMIC List 2020 noted that only 9 (20%) of 45 articles reported vendors as having adequate food safety knowledge. Maintaining personal hygiene is crucial in all aspects of food handling since the human body can be a source of contamination.

The findings of this study indicated that raw meat handlers exhibited inadequate personal hygiene practices. Although most respondents practised personal hygiene, such as washing hands before touching meat and keeping fingernails short, it is not enough when some aspects of personal hygiene related to meat handling are omitted. For instance, none of the respondents used gloves while handling meat, and most did not wear hair caps, nose masks, or waterproof footwear. A similar study conducted by Sani and Siow [34] supported these findings, with meat handlers admitting to not using gloves, which was also confirmed by Elneim [35]. In contrast, Addison [36] reported a significantly higher percentage of food handlers who utilized gloves, hair caps, and aprons, contradicting the results of this study. Microorganisms such as *Escherichia coli* and *Salmonella* can persist on the fingertips, posing a risk of contamination. According to Addison [36], all people who handle food wash their hands before doing so. The results are similar to this study, in which participants who handled raw meat washed their hands frequently using soap both before and after touching the meat and persons with injuries are not permitted to touch meat. These findings align with the research conducted by Sani and Siow [34], which revealed that most meat handlers (93.2%) were aware of the risks associated with handling meat using injured hands.

The study also indicated that raw meat sold in retail settings is highly vulnerable to microbial contamination. These findings are similar to those reported by Olu-Taiwo [37] and Adjei [38] in Accra and Ashaiman, Ghana, respectively, where they found that most beef sold was contaminated with coliform and pathogenic bacteria. Similar findings have been reported in China [39], Ethiopia [40, 41], Pakistan [42], and Nigeria [43]. The meat contamination by microbes can occur at various stages; during slaughter, processing, and transportation. It is vital to improve the meat handling and processing chain to safeguard public health against the risks of foodborne bacterial infections [40]. Microbial populations in meat pose a significant challenge for the meat industry [44]. The results of the microbial assessment showed elevated levels of microbial counts in the raw beef samples, indicating contamination of the beef. Potential sources of contamination could include cutting knives, containers, intestinal contents, water, hides, meat handlers, and the environments involved in meat processing and selling. This study also demonstrated that the samples were contaminated with different types of bacteria, with *Staphylococcus* spp. and *E. coli* being the most prevalent. *Staphylococcus* spp. was present in all of the samples, consistent with the findings of other studies, which also observed a high occurrence of *Staphylococcus aureus* in raw meats [37]. *Staphylococcus aureus* can survive in diverse environments (dry and stressful environments) and this could favor the growth of the organism in many food products [45]. This significant prevalence of *Staphylococcus* species suggests the possibility of contamination from the people who handle the raw meat. Poor hygiene practices among personnel in the meat industry, as well as inadequate sanitation measures, contribute to this issue. This study did not confirm the presence of the isolated microbes. However, the identified microorganisms provide insight into the safety of meat on the market.

The microbial variety found in samples obtained from retail shops in this study indicates unsatisfactory sanitary conditions throughout the handling, processing, and transporting of carcasses for sale. This finding is similar to previous studies [46, 47], which identified gram-negative bacteria such as *E. coli* in the meat samples. Additionally, *Staphylococcus* species were also identified from the meat samples. These organisms have been previously linked to different infectious and foodborne disease outbreaks in Ghana [48]. *Staphylococcus aureus*, a bacteria typically found in the body, indicates contamination by handlers. This bacterium can be transferred to food during various stages, such as harvesting, processing, and storage [49]. *Staphylococcus aureus* is a cause of food poisoning called staphylococcal food poisoning, characterized by symptoms like diarrhea and vomiting caused by consuming an enterotoxin produced by the bacteria [50]. It is important to note that detecting *E. coli* in the samples is a strong indication of fecal contamination, as this bacterium naturally resides in the intestinal tract. This contamination can occur due to unsanitary conditions during meat processing, such as unclean water supply, unsterilized utensils, and exposure to flies. *E. coli* can cause gastroenteritis, particularly in infants and young children [51]. According to Lianou [52], proper cooking methods and hygiene practices can significantly reduce the presence of harmful microorganisms in fresh meat.

## Conclusion

The findings of this study successfully addressed the initial objective, which aimed to evaluate the food safety knowledge among people who handle raw meat. While the results indicated a need for more adequate understanding regarding foodborne pathogens and high-risk groups, some positive responses were obtained from the people who handle raw meat. Regarding the second objective, which sought to assess the food safety practices among raw meat handlers in the butchery shops of Kumasi Meat Abbatoir, the results revealed that the meat handlers exhibited inadequate food safety practices. Approximately half of them admitted to occasionally handling meat with sores on their hands, and they also reported using the same towel for cleaning purposes at the workplace.

The microbial quality of fresh beef available in the area was examined in this study, revealing contamination with *Staphylococcus* spp. and *Escherichia coli*. The overall sanitary conditions observed at the slaughterhouse and meat shops, as well as the poor hygienic practices of the butchers, were identified as significant factors contributing to the microbial contamination of the beef. These microorganisms in raw beef pose a potential public health hazard. It serves as a warning sign for the possible occurrence of foodborne intoxication and infection if proper control measures are not implemented. There is a need to educate meat handlers and the general public on food safety to ensure that the food consumed is free from contamination. Additionally, agencies responsible for food safety should intensify the monitoring and inspection of meat processing facilities to enforce and maintain sanitary standards among raw meat handlers. This could reduce the outbreak of diseases associated with consuming contaminated foods and contribute to the achievement of the sustainable development goals, especially goal 3 (good health and wellbeing).

## Data Availability

All data generated or analysed during this study are included in this manuscript.

## Abbreviations

ANOVA: Analysis of Variance
BGA: Brilliant Green Agar
*E. coli*: *Escherichia coli*
HACCP: Hazard Analysis Critical Control Points
KACL: Kumasi Abattoir Company Limited
KNUST: Kwame Nkrumah University of Science and Technology
PCA: Plate Count Agar
RVB: Rappaport Vassiliadis Broth
SMA: Salt Mannitol Agar
SPSS: Statistical Package for the Social Sciences
TEC: Total *Escherichia coli* Count
TSC: Total Staphylococcus Count
TSSC: Total *Salmonella* Count
TVC: Total Viable Counts
WHO: World Health Organization
XLD: Xylose Lysine Deoxycholate

## References

[1] Gizaw Z (2019). Public health risks related to food safety issues in the food market: a systematic literature review. Environ Health Prev Med.

[2] Dapuliga CC, Balali GI, Titus OO, Osafo R, Taufiq M (2022). Food safety in sub-Sahara Africa, an insight into Ghana and Nigeria. Environ Health Insights.

[3] Xiong Y, Li W, Liu T (2020). Risk early warning of food quality safety in meat processing industry. Int J Environ Res Public Health.

[4] WHO. Food safety. 2022. Retrieved at https://www.who.int/news-room/fact-sheets/detail/food-safety#:~:text=Children%20under%205%20years%20of,national%20economies%2C%20tourism%20and%20trade. Accessed 31 Oct 2023.

[5] WHO. First-ever world food safety day elevates attention to dangerous foodborne diseases in Africa. 2019. Retrieved at https://www.afro.who.int/news/first-ever-world-food-safety-day-elevates-attention-dangerous-foodborne-diseases-africa. Accessed 31 Oct 2023.

[6] Haileselassie M, Taddele H, Adhana K, Kalayou S (2013). Food safety knowledge and practices of abattoir and butchery shops and the microbial profile of meat in Mekelle City, Ethiopia. Asian Pac J Trop Biomed.

[7] Kagambega A, Haukka K, Siitonen A, Traore AS, Barro N (2011). Prevalence of *Salmonella enterica* and the hygienic indicator Escherichia Coli in raw meat at markets in Ouagadougou, Burkina Faso. J Food Prot.

[8] Akabanda F, Hlortsi EH, Owusu-Kwarteng J (2017). Food safety knowledge, attitudes and practices of institutional food-handlers in Ghana. BMC Public Health.

[9] Parry-Hanson Kunadu A, Ofosu DB, Aboagye E, Tano-Debrah K (2016). Food safety knowledge, attitudes and self-reported practices of food handlers in institutional foodservice in Accra, Ghana. Food Control.

[10] Manko NA, Abor PA (2023). Hygienic practices of food vendors in food safety on the University of Ghana campus. J Food Saf Hygiene.

[11] Rheinländer T, Olsen M, Bakang JA, Takyi H, Konradsen F, Samuelsen H (2008). Keeping up appearances: perceptions of street food safety in Urban Kumasi, Ghana. J Urb Health.

[12] Dun-Dery EJ, Addo HO (2016). Food hygiene awareness, processing and practice among street food vendors in Ghana. Food Public Health.

[13] Addo-Tham R, Appiah-Brempong E, Vampere H, Acquah-Gyan E, Gyimah Akwasi A (2020). Knowledge on food safety and food-handling practices of street food vendors in Ejisu-Juaben Municipality of Ghana. Adv Public Health.

[14] Al Banna MH, Disu TR, Kundu S, Ahinkorah BO, Brazendale K, Seidu A-A (2021). Factors associated with food safety knowledge and practices among meat handlers in Bangladesh: a cross-sectional study. Environ Health Prev Med.

[15] Augustin JC, Kooh P, Bayeux T, Guillier L, Meyer T, Jourdan-Da Silva N (2020). Contribution of foods and poor food-handling practices to the burden of foodborne infectious diseases in France. Foods.

[16] WHO. Promoting food safety in Ghana through community engagement. 2023. Retrieved at https://www.afro.who.int/photo-story/promoting-food-safety-ghana-through-community-engagement#:~:text=In%20Ghana%2C%20foodborne%20diseases%20such,levels%20of%20literacy%20and%20education. Accessed 15 Jan 2024.

[17] Osei-Tutu B, Anto F (2016). Trends of reported foodborne diseases at the Ridge Hospital, Accra, Ghana: a retrospective review of routine data from 2009–2013. BMC Infect Dis.

[18] Twum E (2016). Microbial quality of fresh beef sold in the Birim North District of the Eastern Region of Ghana.

[19] Di Novi C, Marenzi A (2022). Improving health and sustainability: patterns of red and processed meat consumption across generations. Health Policy.

[20] Salam N (2016). Microbial content of meat and poultry product sold at Kumasi Central Market, Ghana.

[21] Milford AB, Le Mouël C, Bodirsky BL, Rolinski S (2019). Drivers of meat consumption. Appetite.

[22] Quartey JN. Factors associated with food safety practices among raw meat handlers in Turaku slaughter slab and Madina market in Accra, Ghana. Masters' Dissertation, University of Ghana; 2018.

[23] Frimpong S, Gebresenbet G, Bosona T, Bobobee E, Aklaku E (2012). Animal supply and logistics activities of abattoir chain in developing countries: the case of Kumasi Abattoir, Ghana.

[24] Gomes-Neves E, Cardoso CS, Araújo AC, da Costa JMC (2011). Meat handlers training in Portugal: a survey on knowledge and practice. Food Control.

[25] Mazizi B, Muchenje V, Makepe M, Mutero G (2017). Assessment of aerobic plate counts, Staphylococcus aureus, Escherichia coli and Salmonella in meat sold by street vendors in the Eastern Cape Province, South Africa. J Food Nutr Res.

[26] Habib F, Rind R, Durani N, Bhutto AL, Buriro RS, Tunio A (2015). Morphological and cultural characterization of Staphylococcus aureus isolated from different animal species. J Appl Environ Biol Sci.

[27] Ansari-Lari M, Soodbakhsh S, Lakzadeh L (2010). Knowledge, attitudes and practices of workers on food hygienic practices in meat processing plants in Fars, Iran. Food Control.

[28] Gaungoo Y, Jeewon R (2013). Effectiveness of training among food handlers: a review on the Mauritian framework. Curr Res Nutr Food Sci J.

[29] Makhunga SE, Margaret M, Khumbulani H (2023). Food handlers’ knowledge, attitudes and self-reported practices regarding safe food handling in charitable food assistance programmes in the eThekwini District, South Africa: cross-sectional study. BMJ Open.

[30] Tokuç B, Ekuklu G, Berberoğlu U, Bilge E, Dedeler H (2009). Knowledge, attitudes and self-reported practices of food service staff regarding food hygiene in Edirne, Turkey. Food Control.

[31] Yenealem DG, Yallew WW, Abdulmajid S (2020). Food safety practice and associated factors among meat handlers in Gondar town: a cross-sectional study. J Environ Public Health.

[32] Tamiru S, Bidira K, Moges T, Dugasa M, Amsalu B, Gezimu W (2022). Food safety practice and its associated factors among food handlers in food establishments of Mettu and Bedelle towns, Southwest Ethiopia, 2022. BMC Nutr.

[33] Wallace F, Mittal N, Lambertini E, Nordhagen S (2022). Vendor knowledge, attitudes, and practices related to food safety in low- and middle-income countries: a scoping review. J Food Prot.

[34] Sani NA, Siow ON (2014). Knowledge, attitudes and practices of food handlers on food safety in food service operations at the Universiti Kebangsaan Malaysia. Food Control.

[35] Elneim EAA (2013). Practice in the preparation, handling and storage of street food vendors women in Sinja City (Sudan). Literacy.

[36] Addison I. Hygienic practices among food vendors in the University of Ghana. Unpublished doctoral dissertation, University of Ghana. 2015.

[37] Olu-Taiwo M, Obeng P, Forson AO (2021). Bacteriological analysis of raw beef retailed in selected open markets in Accra, Ghana. J Food Qual.

[38] Adjei VY, Mensah GI, Parry-Hanson Kunadu A, Tano-Debrah K, Ayi I, Addo KK (2022). Microbial safety of beef along beef value chains in the Ashaiman Municipality of Ghana. Front Vet Sci.

[39] Yang S, Pei X, Yang D, Zhang H, Chen Q, Chui H (2018). Microbial contamination in bulk ready-to-eat meat products of China in 2016. Food Control.

[40] Kanko T, Seid M, Alemu M (2023). Evaluation of bacteriological profile of meat contact surfaces, handling practices of raw meat and its associated factors in butcher shops of Arba Minch town, southern Ethiopia-a facility based cross sectional study. Food Saf Risk.

[41] Mustefa WS (2021). Microbiological evaluation of meat sold in butcheries shop of Cheleleka town in anchar woreda, West Harerge, Oromia, Ethiopia Western Ethiopia. J Food Sci Nutr Therapy.

[42] Ansari S, Abro S, Tanweer A, Sethar A, Abbas G, Ansari S (2022). Evaluation of bacterial contamination from raw and cooked fish, mutton and beef sold by local vendors in Hyderabad, Pakistan. J Anim Health Prod.

[43] Adesokan HK, Obimdike OC, Adetunji VO. Informal and formal meat marketing in Ibadan, Nigeria: public health implications from microbial assessment. PAMJ-One Health. 2021;5(15).

[44] Komba EV, Komba EV, Mkupasi EM, Mbyuzi AO, Mshamu S, Mzula A (2012). Sanitary practices and occurrence of zoonotic conditions in cattle at slaughter in Morogoro Municipality, Tanzania: implications for public health. Tanzan J Health Res.

[45] Kadariya J, Smith TC, Thapaliya D (2014). Staphylococcus aureus and staphylococcal food-borne disease: an ongoing challenge in public health. BioMed Research International.

[46] Omorodion N, Odu N (2014). Microbiological quality of meats sold in Port Harcourt Metropolis, Nigeria. Nat Sci.

[47] Adzitey F, Ahmed AA, Moses O (2014). Microbial quality of beef in the Yendi Municipality of Ghana. Global J Anim Sci Res.

[48] Mingle CL, Darko G, Borquaye LS, Asare-Donkor NK, Woode E, Koranteng F (2021). Veterinary drug residues in beef, chicken, and egg from Ghana. Chem Afr.

[49] Eze V, Ivuoma N (2012). Evaluation of microbial quality of fresh goat meat sold in Umuahia market, Abia State, Nigeria. Pakistan J Nutr.

[50] Hennekinne J-A, De Buyser M-L, Dragacci S (2012). Staphylococcus aureus and its food poisoning toxins: characterization and outbreak investigation. FEMS Microbiol Rev.

[51] Gwimbi P, George M, Ramphalile M (2019). Bacterial contamination of drinking water sources in rural villages of Mohale Basin, Lesotho: exposures through neighbourhood sanitation and hygiene practices. Environ Health Prev Med.

[52] Lianou A, Panagou EZ, Nychas GJE (2023). Meat safety—I foodborne pathogens and other biological issues.

